# Formation of Apatite Coatings on an Artificial Ligament Using a Plasma- and Precursor-Assisted Biomimetic Process

**DOI:** 10.3390/ijms140919155

**Published:** 2013-09-17

**Authors:** Hirotaka Mutsuzaki, Yoshiro Yokoyama, Atsuo Ito, Ayako Oyane

**Affiliations:** 1 Department of Orthopaedic Surgery, Ibaraki Prefectural University of Health Sciences, 4669-2 Ami Ami-machi, Inashiki-gun, Ibaraki 300-0394, Japan; E-Mail: mutsuzaki@ipu.ac.jp; 2 Nanosystem Research Institute, National Institute of Advanced Industrial Science and Technology (AIST), Central 4, 1-1-1, Higashi, Tsukuba-shi, Ibaraki 305-8562, Japan; E-Mail: yokoyama.yj@om.asahi-kasei.co.jp; 3 Human Technology Research Institute, National Institute of Advanced Industrial Science and Technology (AIST), Central 6, 1-1-1, Higashi, Tsukuba-shi, Ibaraki 305-8566, Japan; E-Mail: atsuo-ito@aist.go.jp

**Keywords:** plasma surface modification, alternate dipping treatment, apatite, simulated body fluid (SBF), artificial ligament

## Abstract

A plasma- and precursor-assisted biomimetic process utilizing plasma and alternate dipping treatments was applied to a Leed-Keio artificial ligament to produce a thin coating of apatite in a supersaturated calcium phosphate solution. Following plasma surface modification, the specimen was alternately dipped in calcium and phosphate ion solutions three times (alternate dipping treatment) to create a precoating containing amorphous calcium phosphate (ACP) which is an apatite precursor. To grow an apatite layer on the ACP precoating, the ACP-precoated specimen was immersed for 24 h in a simulated body fluid with ion concentrations approximately equal to those in human blood plasma. The plasma surface modification was necessary to create an adequate apatite coating and to improve the coating adhesion depending on the plasma power density. The apatite coating prepared using the optimized conditions formed a thin-film that covered the entire surface of the artificial ligament. The resulting apatite-coated artificial ligament should exhibit improved osseointegration within the bone tunnel and possesses great potential for use in ligament reconstructions.

## 1. Introduction

Artificial ligaments are commonly used in ligament operations, including anterior cruciate ligament (ACL) reconstructions, patella tendon repair, and rotator cuff repair, in which the artificial ligaments are inserted in bone tunnels. In particular, the Leeds-Keio artificial ligament (Xiros plc., Leeds, UK), which is composed of poly(ethylene terephthalate) (PET), has been widely used in clinical treatments [1,2]. ACL reconstructions performed using hamstring tendons fixed with staples via Leeds-Keio ligaments in the tibia are called suspensory fixations [1,2]. Because the fixation sites between the soft tissue and the hard tissue are mechanically the weakest regions during the early postoperative period [3,4], the rapid and robust osseointegration of artificial ligaments within bone tunnels is critical for successful ligament reconstruction.

In 2011, Li *et al.* demonstrated that apatite coatings on the surfaces of artificial ligaments are effective in enhancing the osseointegration of artificial ligaments within the bone tunnel [5]. This effect was enabled because apatite is a major inorganic component of natural bone and exhibits good biocompatibility and osteoconductivity [6,7]. In Li’s study, an apatite coating was produced on an artificial ligament using plasma surface modification and subsequent immersion in a solution containing apatite powders. The resulting coating comprised micro-sized apatite powders dispersed throughout the ligament surface; however, the majority of the ligament surface remained uncoated. A thin-film apatite coating over the entire surface of a ligament (insertion site) should be beneficial in further enhancing osseointegration within the bone tunnel [8].

Among the various apatite-based thin-film coating techniques, biomimetic processes that employ a simulated body fluid (SBF) [9,10] as the coating solution are especially advantageous in producing bone-like apatite and are even compatible with soft devices composed of low-melting-point polymers [11–13]. Recently, we developed an advanced biomimetic process [13–18] involving a simplified alternating dipping treatment [19]. In this process, a substrate material is precoated with amorphous calcium phosphate (ACP), which is an apatite precursor, using a simplified alternate dipping treatment followed by immersion in SBF [20]. A continuous, thin coating layer of apatite forms on the ACP-precoated substrate within 24 h in SBF. This apatite coating technique, also known as an ACP-assisted biomimetic process [13,21], provides the advantage of simplicity compared with previous biomimetic processes, which require the use of carboxymethylation [22], photografting [23], or sol-gel methods [24,25].

In the present study, we applied the ACP-assisted biomimetic process to a Leeds-Keio artificial ligament to prepare a thin-film apatite coating for improved osseointegration. To optimize the coating conditions, we first prepared hot-pressed plate specimens from Leeds-Keio artificial ligament meshes. The plate specimens were then subjected to plasma surface modification [15–18,26] under various conditions, followed by the alternate dipping treatment and SBF immersion. The quality of the apatite coating on the specimen and the coating adhesion to the specimen surface were evaluated as a function of the conditions (plasma power density) of the plasma surface modification. The resulting optimized coating process was then applied to a Leeds-Keio artificial ligament mesh to demonstrate its potential application in ligament operations.

## 2. Results

### 2.1. Surface Structural Changes due to Plasma Treatment

**Figure 1:**
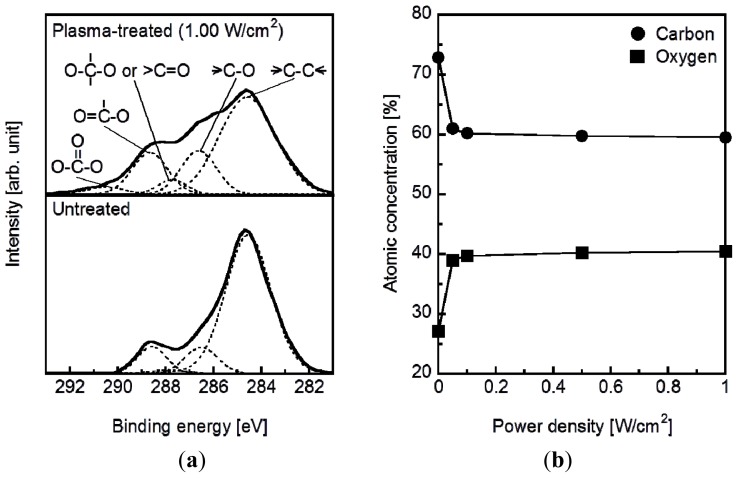
(**a**) C_1S_ XPS spectra and (**b**) the surface atomic concentrations of C and O on plasma-treated (0.05–1.00 W/cm^2^) and untreated plate specimens.

The plasma surface modification introduced oxygen-containing functional groups to the specimen surface according to the results of X-ray photoelectron spectroscopy (XPS). Figure 1a displays the C_1s_ XPS spectra of the surfaces of the plasma-treated (1.0 W/cm^2^) and untreated plate specimens. The C_1s_ peaks (solid line) from the plasma treated and untreated specimens were resolved into five peaks (dotted line), which were attributed to the carbon atoms in –C–C–, –C–O–, –C=O/–O–C–O–, –O–C=O, and –O–C(=O)–O– [27,28]. The peak intensity of the methylene carbon decreased and the intensities of all of the other peaks increased following the plasma treatment. This result can be attributed to the cleavage of the methylene and ester linkages on the specimen surface to produce oxygen-containing functional groups such as hydroxyl, carbonyl, and carboxyl groups. According to the surface composition analysis, the density of the oxygen-containing functional groups on the specimen surface increased with increasing plasma power density up to 0.1 W/cm^2^ and saturated at higher plasma power densities (Figure 1b).

**Figure 2:**
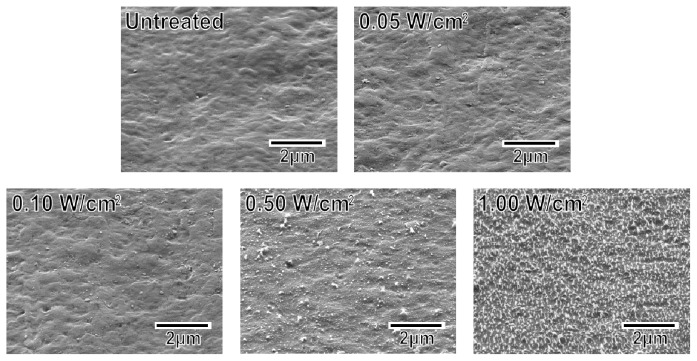
SEM images of the surfaces of the untreated and plasma-treated (0.05–1.00 W/cm^2^) plate specimens.

The surface roughness of the specimens increased with increasing plasma power density. As displayed in the scanning electron microscopy (SEM) images in Figure 2, the untreated specimen and the specimens treated at relatively low plasma power densities (0.05 and 0.10 W/cm^2^) exhibited smooth surfaces. The specimens treated at higher plasma power densities (0.50 and 1.00 W/cm^2^) exhibited surfaces with submicron-sized granules. In addition, the granules on the specimen surface increased in number density with increasing plasma power density. These granules most likely formed via the cleavage of the methylene and ester linkages at the specimen surface and the subsequent etching of the degraded portion during the plasma treatment [29].

### 2.2. Surface Structural Changes due to the Alternate Dipping Treatment

**Figure 3:**
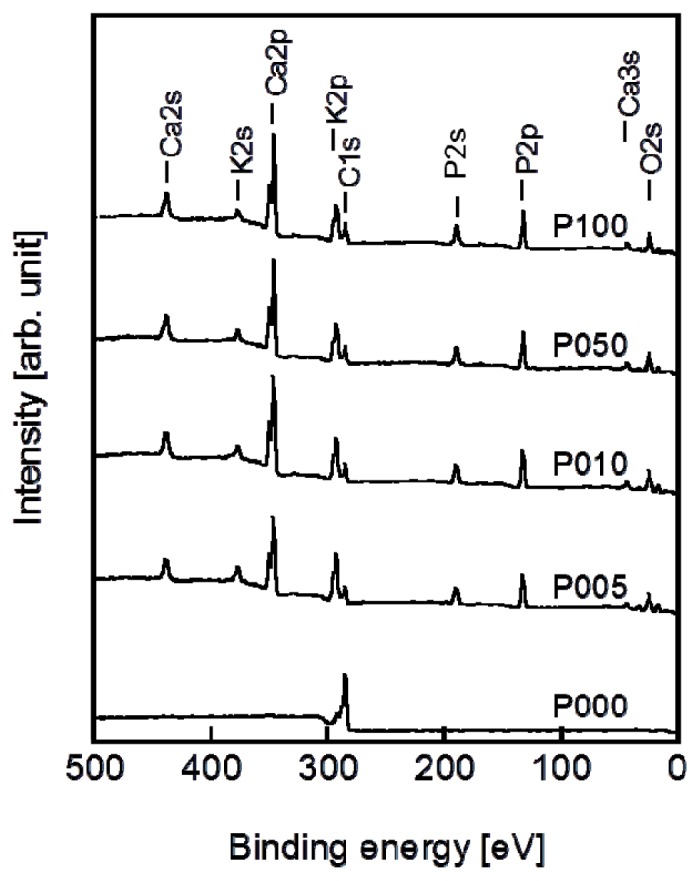
XPS spectra of the surfaces of the plate specimens P000, P005, P010, P050, and P100.

**Figure 4:**
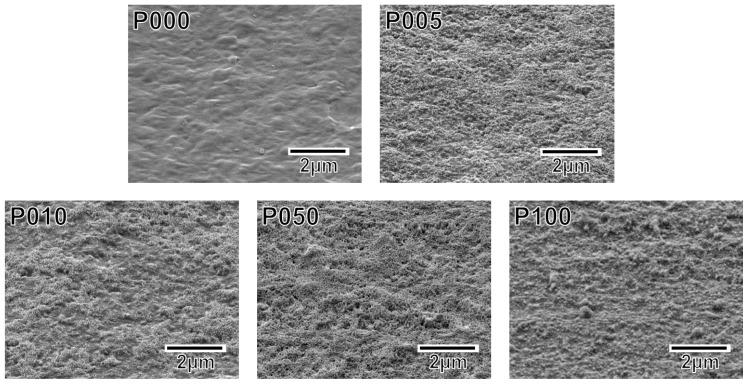
SEM images of the surfaces of the plate specimens P000, P005, P010, P050, and P100.

Following the alternate dipping treatment, an ACP precoating formed on the plasma-treated plate specimens (P005, P010, P050, and P100) but not on the untreated plate specimen (P000). According to the XPS results, calcium phosphate was deposited on the surfaces of all of the plate specimens except for P000 (Figure 3). According to our previous transmission electron microscopy results, the calcium phosphate was most likely in the form of ACP (20). The ACP precoating slightly altered the morphology of the specimen surfaces, as revealed in the SEM images in Figure 4. The ACP precoatings were apparently uniform and covered the entire surfaces of the specimens.

### 2.3. Surface Structural Changes due to SBF Immersion

**Figure 5:**
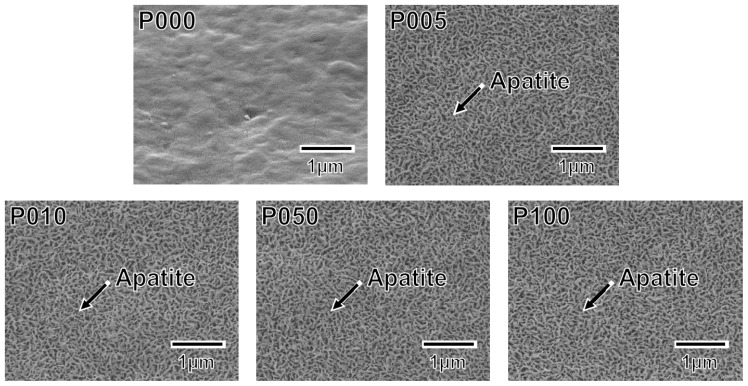
SEM images of the surfaces of the plate specimens P000, P005, P010, P050, and P100 following immersion in SBF for 24 h.

**Figure 6:**
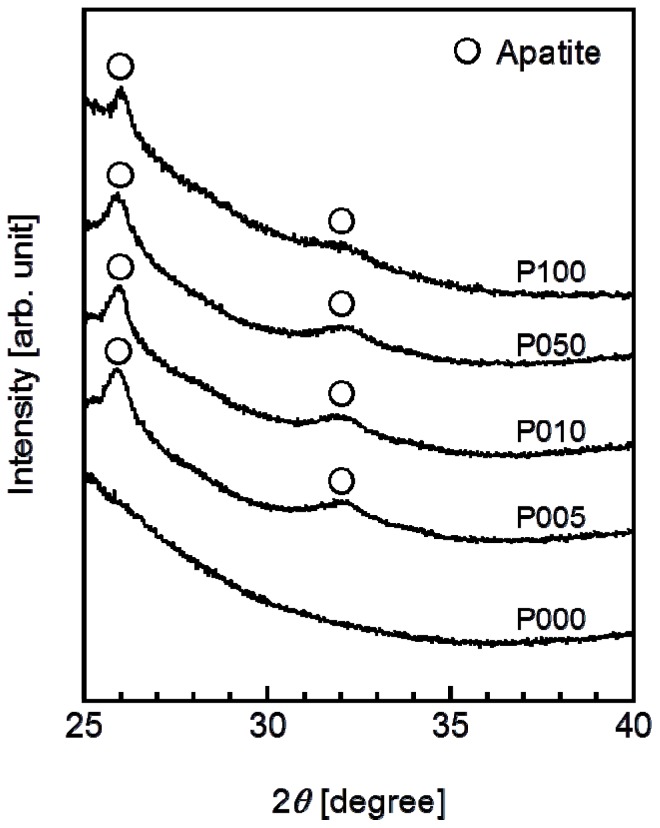
TF-XRD patterns from the surfaces of the plate specimens P000, P005, P010, P050, and P100 following immersion in SBF for 24 h.

A thin coating of apatite formed on the ACP-precoated plate specimens (P005, P010, P050, and P100) following a 24 h immersion in SBF (Figure 5). The apatite coating exhibited a nano-porous structure and covered the entire surfaces of the specimens. According to the results of thin-film X-ray diffractometry (TF-XRD), this coating layer was composed of low-crystalline apatite [30] (Figure 6). On the P000 plate specimen without the ACP precoating, no coating layer was observed following the immersion in SBF (Figures 5 and 6). These results correspond well with our previous results from other polymer substrates [15–18,26].

### 2.4. Adhesion Strength of the Apatite Coating to the Specimen

**Figure 7:**
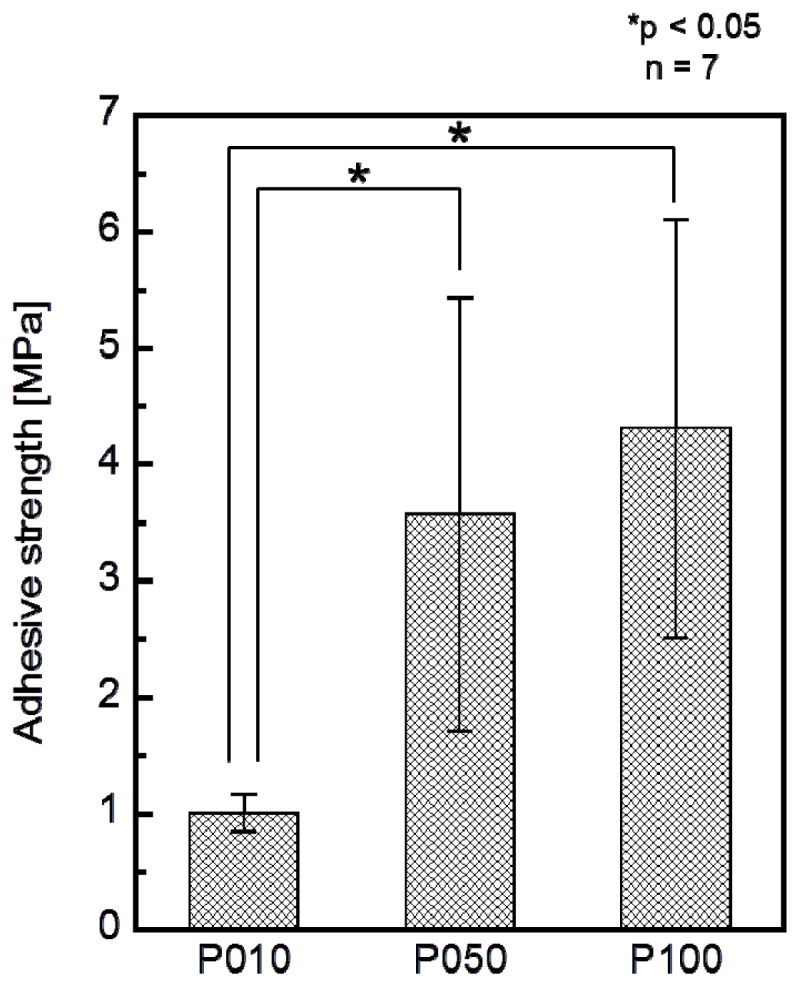
Adhesion strengths of the apatite coatings to the surfaces of the plate specimens P010, P050, and P100 (*n* = 7, *p < 0.05).

**Figure 8:**
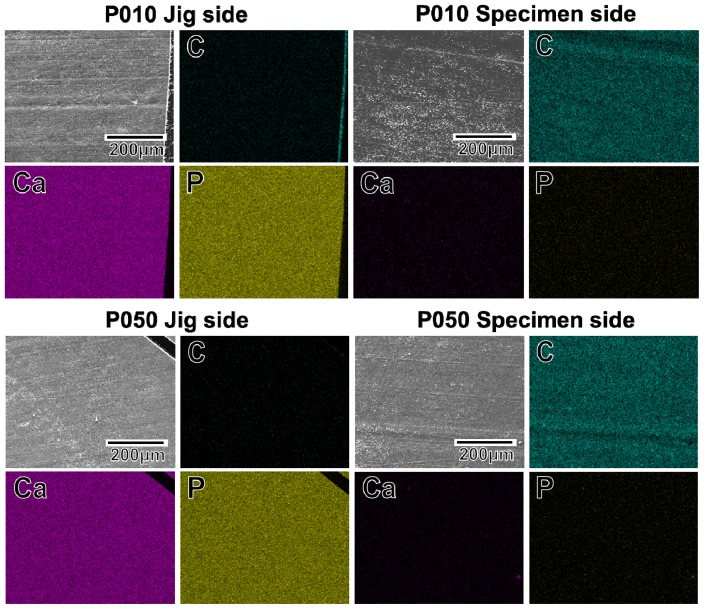

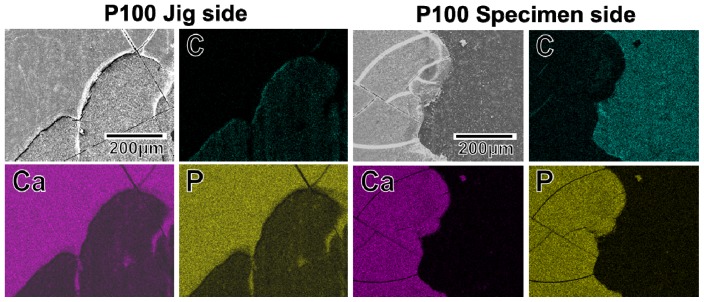
SEM (upper left image in a set of 4 images) and EDX (C, Ca, P) images of the specimen side and jig side fractured surfaces, after the measurement of adhesion strength of the apatite coatings to the surfaces of the plate specimens P010, P050, and P100.

The adhesion strength of the apatite coating to the plate specimen improved with the use of increasing plasma power density for the plasma surface modification. P005 was excluded from the adhesion strength measurement based on the results of a screening test in which the coating layer detached from the specimen surface using a Scotch^®^ tape-detachment test. The apatite coatings on the plate specimens treated with higher plasma powers (P010, P050, and P100) remained adhered even after the tape-detachment test and were therefore subjected to the quantitative adhesion strength-measurement. It can be observed in Figure 7 that the adhesion strengths of the apatite coatings to the P050 and P100 surfaces were significantly higher than that to the P010 surface (*p* = 0.0015 and *p* < 0.0001, respectively). The average adhesion strength of the apatite coating to the P100 surface was higher than that to the P050 surface, although the difference was not statistically significant. According to the energy dispersive electron probe X-ray analysis (EDX) of the fractured surfaces, the fracture occurred at the coating-specimen interface for P010 and P050. For these specimens, Ca and P, which are component elements of apatite, were detected by EDX on the jig side, whereas they were not detected on the specimen side (Figure 8). The fracture in P100 occurred not only at the coating–specimen interface but also at the coating–glue interface. In the case of P100, a splintered layer composed of Ca and P was observed on both the jig and the specimen sides (Figure 8).

### 2.5. Application to Artificial Ligament Meshes

**Figure 9:**
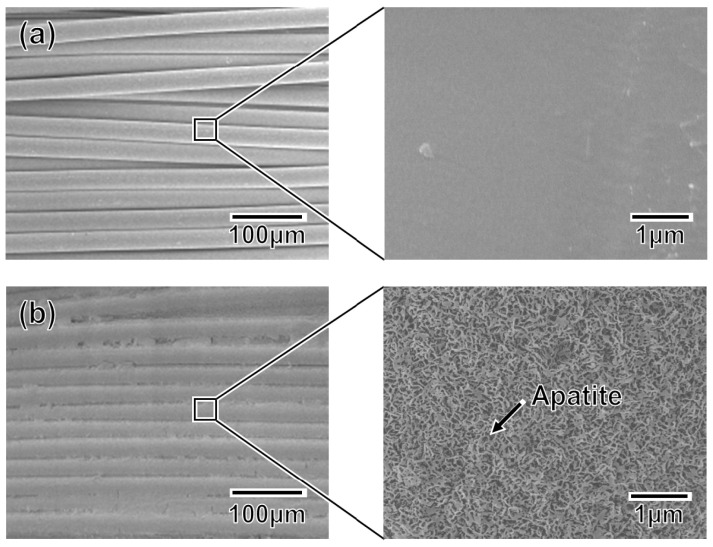
SEM images collected at varying magnifications from (**a**) the untreated mesh specimen and (**b**) the P100 mesh specimen after a 24 h immersion in SBF.

An apatite coating layer was successfully deposited on the mesh specimen as well as on the plate specimens. Figure 9 displays SEM images of the surfaces of (a) the untreated (as-prepared) mesh specimen and (b) the P100 plasma-treated mesh specimen following a 24 h immersion in SBF. As displayed in Figure 9b, the individual fibers composing the artificial ligament were fully coated with a continuous thin-film apatite layer exhibiting a nano-porous structure similar to that formed on the plate specimens (see P100 in Figure 5).

## 3. Discussion

The developed plasma- and ACP-assisted biomimetic process was found to be effective in forming apatite layers on Leeds-Keio artificial ligaments (Figure 9). Because of its similarities to other hydrophobic polymers [15–18,26], plasma surface modification was necessary to allow the formation of an apatite coating on the artificial ligament (see Figures 5 and 6). A mechanism for the apatite formation in the ACP-assisted biomimetic process was proposed in our previous reports [15–17]. Briefly, the apatite precursor ACP was precoated on the plasma-treated specimen using an alternate dipping treatment that takes advantage of the anchoring effect of the oxygen-containing functional groups on the specimen surface [20]. When the ACP-precoated specimen was subsequently immersed in SBF, the ACP on the specimen surface promoted the nucleation and formation of calcium phosphates. With increasing immersion duration in SBF, the ACP and/or other metastable calcium phosphates were eventually converted to apatite, which is the most stable crystalline phase in a neutral solution [31]. Following the 24 h immersion in SBF, a continuous thin-film coating of apatite that was similar in composition and crystal structure to bone apatite [32] formed over the entire surface of the specimen. In contrast, without the plasma treatment, the ACP precoating was unable to form during the alternate dipping treatment because the specimen surface was hydrophobic and its wettability with aqueous solutions was insufficient. Without the ACP precoating, no apatite layer was able to grow on the specimen surface, even after 24 h in SBF.

As reported for other polymers, including poly(l-lactic acid) [16] and ethylene-vinyl alcohol copolymer (EVOH) [17], increasing the plasma power density used for the plasma surface modification increased the adhesion of the coating to the specimen surface (Figure 7). Because the density of the oxygen-containing functional groups on the specimen surface was comparable for all power densities from 0.05 W/cm^2^ (P005) to 1.00 W/cm^2^ (P100) (Figure 1b), the increase in surface roughness (Figure 2) may have been the controlling factor enhancing the coating adhesion to the specimen through a mechanical interlocking effect. It should be noted that the optimal plasma power density depended on the type of polymer in the substrate, *i.e.*, 1.50 W/cm^2^ for EVOH [17] and 1.00 W/cm^2^ for poly(l-lactic acid) [16] and the artificial ligament (PET) used in this study. This dependence on polymer identity may have arisen due to the differences in the stability of the polymer main chains: the ethylene linkages in EVOH are relatively stable compared with the ester linkages in poly(l-lactic acid) and PET.

Clinically, the present apatite coating technique employing a plasma- and ACP-assisted biomimetic process would be useful for ligament reconstructions. The shear stress at the bone-graft interface associated with the bungee effect (longitudinal micromotion) [33] was found to promote loose, fibrous bonding in previous reports [4,8]. Moreover, enlargement of the articular end of the bone tunnel is a common problem in ACL reconstruction [34] because the graft-tunnel motion can be greater at the tunnel aperture site than at the extra-articular end of the tunnel for grafts fixed by suspensory fixation. It has been reported that even a powdery apatite coating, in which micro-sized apatite powders are dispersed randomly over an artificial ligament, is effective in enhancing artificial ligament osseointegration within the bone tunnel [5]. The apatite coating prepared by the present plasma- and ACP-assisted biomimetic process was in the form of a thin-film and covered the entire surface of the artificial ligament (Figure 9). This coating layer adhered to the specimen surface so strongly that, in tensile tests, fracture occurred not only at the coating–specimen interface but also at the coating–glue interface. Furthermore, the apatite grown in SBF is low-crystalline and calcium deficient apatite containing carbonate, sodium, and magnesium ions (Ca/P = 1.51, Mg/Ca = 0.04, Na/Ca = 0.03, carbonate content = 2.64 wt%, lattice constant *a* = 9.432 Å, *c* = 6.870 Å [32]), and is similar to bone apatite in both composition and crystal structure [32]. Such bonelike apatite grown in SBFs exhibits good osseointegration *in vivo* [35,36]. Based on these facts, the apatite-coated artificial ligament prepared by the present process should exhibit improved osseointegration within the bone tunnel. *In vivo* experiments for the present apatite-coated artificial ligament are necessary to evaluate the anchoring strength between the artificial ligament and bone, which is associated with histological observations. Moreover, long-term implantation studies are necessary in the future.

## 4. Materials and Methods

### 4.1. Preparation of Specimens

Hot-pressed plate specimens 1 mm in thickness and 10 mm × 10 mm in size were prepared by hot-pressing Leeds-Keio artificial ligament meshes (Xiros plc., Leeds, UK) at 270 °C followed by cutting. The mesh specimens were obtained by cutting the Leeds-Keio artificial ligament mesh into squares 5 mm × 5 mm in size. Both the plate and mesh specimens were ultrasonically washed with ethanol then dried under vacuum at 60 °C for 24 h.

### 4.2. Plasma Treatment for Surface Modification

The plate and mesh specimens were subjected to oxygen plasma treatment for surface modification [15–18,26]. The plasma treatment was performed in an oxygen gas (99.999%) atmosphere at a pressure of 30 Pa under an electric field operating at 13.56 MHz for 30 s using a compact ion etcher (FA-1, SAMCO Inc., Kyoto, Japan). The plasma power density was varied from 0.05 to 1.00 W/cm^2^.

### 4.3. ACP Precoating Preparation Using an Alternate Dipping Treatment

The untreated and plasma-treated specimens were subjected to an alternating dipping treatment using calcium and phosphate ion solutions [15,17]. The calcium ion solution was prepared by mixing an aqueous 200 mM CaCl_2_ (Nacalai Tesque Inc., Kyoto, Japan) solution and ethanol (Nacalai Tesque Inc., Kyoto, Japan) in a 50:50 volume ratio. The phosphate ion solution was prepared by mixing an aqueous 200 mM K_2_HPO_4_·3H_2_O (Nacalai Tesque Inc., Kyoto, Japan) solution and ethanol in a 50:50 volume ratio. For rinsing the specimen, an ethanol solution was also prepared by mixing ultrapure water and ethanol (Nacalai Tesque Inc., Kyoto, Japan) in a 50:50 volume ratio.

The specimen was initially dipped in 20 mL of the calcium ion solution for 10 s, then dipped in 20 mL of the ethanol solution for 1 s, and finally dried in air for a few minutes. The specimen was subsequently dipped in 20 mL of the phosphate ion solution for 10 s, dipped again in 20 mL of the ethanol solution for 1 s, and finally dried in air for a few minutes. The dipping and withdrawal rates were fixed at 50 cm/min using a linear head motor equipped with a speed controller (Oriental Motor Co, Ltd, Tokyo, Japan). The alternate dipping in calcium ion and phosphate ion solutions was performed three times at room temperature. The same calcium and phosphate ion solutions and the ethanol solution were used for a given specimen throughout the three alternate dipping cycles.

The specimens subjected to the alternate dipping treatment were labeled based on their prior plasma treatment conditions as P000 (no plasma treatment), P005 (0.05 W/cm^2^), P010 (0.10 W/cm^2^), P050 (0.50 W/cm^2^), and P100 (1.00 W/cm^2^).

### 4.4. Formation of Apatite Coating by Immersion in SBF

The specimens prepared in the preceding section were immersed at 36.5 °C in 30 mL of SBF [9,10] with ion concentrations (Na^+^ 142.0 mM, K^+^ 5.0 mM, Mg^2+^ 1.5 mM, Ca^2+^ 2.5 mM, Cl^−^ 147.8 mM, HCO_3_^−^ 4.2 mM, HPO_4_^2−^ 1.0 mM, SO_4_^2−^ 0.5 mM) approximating those of human blood plasma. The SBF was prepared by dissolving NaCl, NaHCO_3_, KCl, K_2_HPO_4_·3H_2_O, MgCl_2_·6H_2_O, CaCl_2_, and Na_2_SO_4_ in ultra-pure water and buffering the solution to pH 7.40 at 36.5 °C using tris(hydroxymethyl)aminomethane (final concentration = 50 mM) and HCl (Nacalai Tesque Inc, Kyoto, Japan) [10].

The surface characterization of the specimens was performed following 24 h immersion in SBF. The adhesion strength of the apatite coatings to the plate specimens was measured after an 8 d immersion in which the spent SBF was replaced with fresh fluid after 4 d. Following its removal from the SBF, the specimen was gently washed with ultrapure water and dried in air at room temperature.

### 4.5. Surface Characterization

The surfaces of the specimens were examined by XPS (Quantum-2000, ULVAC-PHI Inc., Chigasaki, Japan) with Al Kα X-rays, TF-XRD (Rint 2500VH/PC, Rigaku Co., Akishima, Japan) with Cu Kα X-rays, and SEM (XL30, FEI Company Japan Ltd., Tokyo, Japan). The photoelectron take-off angle was set at 45° for XPS. The binding energies measured by XPS were corrected by defining the binding energy of C_1s_ in the CH_2_ group as 284.6 eV. For TF-XRD, the glancing angle of the specimen was set at 1° against the direction of the incident beam.

### 4.6. Measurement of the Adhesion Strength of the Apatite Coating

A 10-μm-thick apatite coating layer was formed on one side only of the plate specimen after an 8 d immersion in SBF (see Section 4.4). Thickness of the coating was confirmed by the method described elsewhere [16,17]. Briefly, only a small area on the surface of the plate specimen was masked with tape during immersion in SBF. After washing and drying of the specimen, the masking tape was detached from the specimen surface and the thickness of the coating formed around the masked area was measured under SEM with the specimen tilt angle set at 45°.

The adhesion strength of the apatite coating to the plate specimen was measured under tensile stress [16,17]. First, both sides of the plate specimen, which was coated on only one side with the 10 μm-thick apatite film, were attached to brazen jigs with a base of 10 mm × 10 mm using a rapid-type Araldite^®^ epoxy resin (Huntsman Co., The Woodlands, TX, USA). The specimen was subsequently left untouched overnight to allow complete solidification of the resin. A tensile load was applied to the specimen using an Instron-type testing machine (UCT-500, Orientec Co. Ltd., Tokyo, Japan) at a crosshead speed of 1 mm/min until fracture occurred. Seven plates were tested for each plasma treatment condition to determine the means and standard deviations of the adhesion strength. Following the test, the fractured surfaces were examined by SEM in combination with EDX (Genesis 2000, AMETEK Co. Ltd., Tokyo, Japan). The measured adhesion strengths were compared using Student’s *t*-test. Significance was considered to be *p* < 0.05.

## 5. Conclusions

A thin coating layer of apatite was successfully formed on a Leeds-Keio artificial ligament using a plasma- and ACP-assisted biomimetic process. Plasma surface modification was essential for producing a successful apatite coating and played an important role in facilitating coating adhesion. The resulting apatite-coated artificial ligament should exhibit improved osseointegration within the bone tunnel and possesses great potential for use in ligament reconstructions.
